# Outcomes of critically ill pregnant COVID-19 patients: a cohort study

**DOI:** 10.5935/2965-2774.20230222-en

**Published:** 2023

**Authors:** Pedro Henrique Rigotti Soares, Cesar Antônio Sebben Filho, Rafaela Doebber Escobar, Leonardo Bianchet Botega, Laura Rodolpho Petry, Wagner Luís Nedel

**Affiliations:** 1 Intensive Care Unit, Grupo Hospitalar Conceição - Porto Alegre (RS), Brazil; 2 Faculdade de Medicina, Universidade do Vale do Rio dos Sinos - São Leopoldo (RS), Brazil

## To the editor,

Information related to coronavirus disease 2019 (COVID-19), caused by severe acute respiratory syndrome coronavirus 2 (SARS-CoV-2), in pregnant women is scarce and limited. Increased oxygen consumption, a reduction in chest wall compliance, and decreased functional residual capacity can exacerbate respiratory distress and may lead to the development of acute respiratory distress syndrome (ARDS).^(1)^ Despite the magnitude of this research question, few studies have reported outcomes in this population.^(2-5)^

This retrospective cohort study included patients admitted to a tertiary intensive care unit (ICU) between June 2020 and May 2021. This study was approved according to national guidelines (Plataforma Brasil 66240017.0.0000.5530). We included patients admitted to the ICU with confirmed SARS-CoV-2 pneumonia. We separately analyzed pregnant patients at any gestational week and the rest of the population of patients admitted to the unit during the same period. Data on the following clinical characteristics were collected: age, sex, comorbidities, Simplified Acute Physiology Score (SAPS 3), and Sequential Organ Failure Assessment (SOFA) score at ICU admission. The primary outcome was the in-hospital mortality rate. The secondary outcome was the number of ventilation-free days (VFDs). The VFDs were defined as the number of days that patients were both alive and free of invasive mechanical ventilation (MV) at 28 and 60 days.

Descriptive statistics included frequencies and percentages for categorical variables and means, standard deviations, confidence intervals, medians, and interquartile ranges for continuous variables. To compare continuous variables, we used the Mann-Whitney test (for independent samples) or Wilcoxon rank-sum test (for paired samples). To analyze categorical variables, we used the chi-squared test. Statistical tests were two-tailed, with significance defined as a p value < 0.05. For all analyses, we used Jamovi 2.3.21.0.

There were 760 ICU patients admitted due to COVID-19, of whom 16 were pregnant women, with a median gestational week of 26.4 weeks (24.3 - 29 weeks). The mortality rate was 25% (four patients). The patients’ clinical characteristics are described in table 1. All pregnant women received dexamethasone. Thirteen patients underwent invasive MV, and 11 of these patients were placed in the prone position, with a median of eight sessions (3 - 9). Five patients required hemodialysis, 13 developed acute respiratory distress syndrome, 9 developed ventilator-associated pneumonia, seven developed catheter-related bloodstream infections and two developed pneumothorax or pneumomediastinum due to barotrauma caused by invasive MV.

**Table 1:** Clinical characteristics of the study cohort

Variable	
Age (years)	32.7 ± 5
SOFA at ICU admission)	3 (2 - 5)
SAPS 3 at ICU admission	48 ± 9
Diabetes	6/16
Hypertension	3/16
Asthma	1/16
Acquired dysfunctions	
ARDS	13/16
Acute kidney injury	9/16
Hemodialysis	5/16
Pneumothorax and pneumomediastinum	2/16
Ventilator-associated pneumonia	9/16
Catheter-related bloodstream infection	7/16
Ventilatory support in ICU	
NIV	10/16
HFNC	1/16
Invasive MV	13/16

SOFA - Sequential Organ Failure Assessment; ICU - intensive care unit; SAPS - Simplified Acute Physiology Score; ARDS - acute respiratory distress syndrome; NIV - noninvasive mechanical ventilation; HFNC - high-flow nasal cannula; MV - mechanical ventilation. Results expressed as mean ± standard deviation, median (interquatile range) or total n/n.

We analyzed the respiratory and ventilatory parameters of pregnant patients who underwent invasive MV at onset. There was no difference between survivors and nonsurvivors regarding the partial pressure of oxygen to the fraction of inspired oxygen (PaO_2_/FiO_2_) ratio (122 [90 - 195] *versus* 126 [100 - 157], respectively, p = 1.0). The pregnant women who survived had increased plateau pressure (30 [30 - 31] cmH_2_O *versus* 26 [24 - 28] cmH_2_O, p = 0.02) and increased driving pressure (17 [16 - 18] cmH_2_O *versus* 13 [13 - 14] cmH_2_O, p = 0.04) compared with those who did not survive. However, there was no difference between survivors and nonsurvivors regarding positive end-expiratory pressure (PEEP) (13.8 [13.3 - 14.5] cmH_2_O *versus* 12.7 [12 - 14] cmH_2_O, p = 0.43). Five patients had a gestational interruption due to refractory hypoxemia, and there was no difference between the PaO_2_/FiO_2_ ratio immediately before and after delivery: 102 (93 - 133) *versus* 86 (58 - 188), respectively), with a mean difference of 16mmHg (95%CI -43 - 75; p = 0.31).

The number of VFDs at 28 days was not different between pregnant and nonpregnant patients (10 days [5 - 15] *versus* 13 days [0 - 20], respectively; p = 0.8). There was also no difference in the number of VFDs at Day 60 between the groups (40 days [34 - 46] in pregnant women *versus* 45 days [29 - 52] in nonpregnant women; p = 1.0). The ICU length of stay of the pregnant patients was 16.5 days (10.8 - 23.3), similar to that of the nonpregnant patients (16 days [10 - 26 days], p = 0.85). The length of hospital stay was also similar between pregnant and nonpregnant patients (31 [22 - 45] days and 31 [19 - 43] days, respectively; p = 0.85). The total invasive MV duration was 16 days (9.5 - 19) in pregnant women and 14 days (8 - 23) in nonpregnant women (p = 0.76).

Our study presents results from a cohort of pregnant women with severe COVID-19 who had a higher mortality rate than those reported in previous studies.^(2-4)^ However, these results may be due to the greater severity of COVID-19 in our population, considering the higher incidence of the need for invasive MV when compared with other cohorts,^(2,5)^ as well as the high incidence of use and large number of prone sessions in patients undergoing invasive MV. This may be due to the greater need for beds during the pandemic, which may have resulted, at least indirectly, in the selection of patients with a higher ventilatory risk for ICU admission, unlike other studies. The ventilatory parameters in this study, however, were similar to those previously reported,^(4)^ corresponding to the expected ventilatory mechanics in these patients with similar gestational weeks.^(2,4,5)^ Our results are in line with previous data in the literature, where no improvement in respiratory variables was found with delivery, whether it was induced or not.^(2,4)^ Data from different cohorts of patients with COVID lead to questions regarding improved outcomes with delivery, and the termination of pregnancy should probably be reserved for specific cases, especially with regard to fetal risk.

Our study found similar outcomes for pregnant patients in terms of MV duration, the number of MV-free days, and the length of stay in the ICU when compared with the general population; however, the small sample size did not allow us to draw definitive conclusions. Another limitation is the lack of a longitudinal assessment of lung mechanics, which prevented us from following the trends in ventilatory parameters and their associations with the outcomes.
